# Long-term accrual of conditions following myocardial infarction: a study of disease trajectories in the Wales Multimorbidity e-Cohort

**DOI:** 10.1186/s12916-025-04520-1

**Published:** 2025-11-26

**Authors:** Jonathan A. Batty, Christopher J. Hayward, Ronan A. Lyons, Chris P. Gale, Niels Peek, Marlous Hall

**Affiliations:** 1 Leeds Institute of Cardiovascular and Metabolic Medicine, University of Leeds, Worsley Building, Level 11, Clarendon Way, Leeds, UK; 2 Leeds Institute for Data Analytics, University of Leeds, Worsley Building, Level 11, Clarendon Way, Leeds, UK; 3 Hull York Medical School, University of York, Heslington, York UK; 4 Academic Cardiovascular Unit, James Cook University Hospital, South Tees NHS Foundation Trust, Middlesbrough, UK; 5 Population Data Science, Faculty of Medicine, Health and Life Science, Swansea University Medical School, Swansea University, Swansea, UK; 6 Department of Cardiology, Leeds General Infirmary, Leeds Teaching Hospital Trust, Leeds, UK; 7 The Healthcare Improvement Studies Institute, University of Cambridge, Cambridge, UK; 8 Division of Informatics, Imaging, and Data Science, School of Health Sciences, University of Manchester, Manchester, UK

**Keywords:** Myocardial infarction, Multimorbidity, Disease trajectories, Clustering, Non-negative matrix factorisation

## Abstract

**Background:**

Improved survivorship following myocardial infarction (MI) has resulted in transferred morbidity to other long-term conditions (LTCs). Understanding of disease accrual over time following MI has been limited by a lack of methodologies that consider real-world complexity. We characterised post-MI multimorbidity trajectories in a real-world population of individuals following MI.

**Methods:**

This population-wide retrospective study comprised all individuals with MI in the Wales Multimorbidity e-Cohort (which included linked primary and secondary care data for 2,902,101 GP-registered residents of Wales; 2005–2019). Single-year post-MI disease clusters and multi-year latent multimorbidity trajectories were constructed from 227 chronic conditions and 62 acute conditions, using non-negative matrix factorisation (NMF). Multinomial logistic regression identified sociodemographic factors associated with single-year post-MI disease clusters. Time-updating flexible parametric survival models quantified the association between multi-year multimorbidity trajectories and long-term all-cause mortality, adjusting for age, sex, year of MI, socioeconomic deprivation and rurality.

**Results:**

In total, 70,529 individuals had an incident MI during the study period (median [interquartile range] age 72 [62–82] years; 40.6% female), with restricted mean post-MI survival of 8.1 years. At MI diagnosis, 67,023 (95.0%) had ≥ 2 LTCs (median 8 [interquartile range; IQR 5–12]), which increased to 99.8% at 1 year (50,633/50,737 surviving patients, median [IQR] 12 [7–17]). NMF classified individuals into one of 10 post-MI disease clusters, based on all observed acute and chronic diagnoses (*n* = 3,954,622) accrued over time. Individuals that followed the most adverse latent multimorbidity trajectory (*n* = 26,035, 36.9%) were characterised by recurrent MI, acute infections and renal disease and had an increased risk of all-cause mortality (adjusted hazard ratio 6.62; 95% CI: 6.09–7.20) compared with individuals in the least adverse trajectory.

**Conclusions:**

Patients with MI have a high pre-existing multimorbidity burden that increases post-MI. Using NMF, we were able to reduce the real-world complexity of all individual diseases accrued over time into 10 latent post-MI multimorbidity trajectories. These trajectories were characterised by specific patterns of acute and chronic conditions, with differential impact on outcomes. These trajectories may enable the implementation of targeted strategies for individuals that are at greatest risk of adverse outcomes.

**Supplementary Information:**

The online version contains supplementary material available at 10.1186/s12916-025-04520-1.

## Background

Multimorbidity (the coexistence of multiple long-term conditions) is a widely recognised global health priority [1–3]. Multimorbidity frequently coexists with polypharmacy, frailty, disability and dependency and is associated with reduced quality of life, increased healthcare utilisation and premature mortality [4]. Despite this, there remains incomplete understanding of the different forms of multimorbidity and the shared biological, environmental and behavioural factors underpinning these. A major barrier to our understanding of multimorbidity is a lack of analytical techniques that account for complex, longitudinal multi-disease interactions, whilst providing output that is interpretable, reproducible and clinically relevant.

Amongst the 1.4 million people in the UK that have survived myocardial infarction (MI), the majority have multimorbidity at diagnosis and are at increased risk of accruing additional long-term health conditions in the post-MI period, compared with age and sex matched peers [5–7]. The improved survivorship in this group observed in recent decades has led to ‘transferred morbidity’ to other long-term conditions [7, 8]. Improved understanding of post-MI multimorbidity trajectories is key to identifying points of intervention, developing preventative strategies and otherwise mitigating the excess risk of adverse clinical outcomes amongst specific patient subgroups [9]. Although studies have described cross-sectional clusters of multimorbidity at MI diagnosis (based on the presence of pre-existing long-term conditions), these do not provide insight into the temporal sequencing of specific conditions (‘multimorbidity trajectories’) over time [6, 10, 11]. To date, only a single study has sought to robustly describe multimorbidity trajectories in the post-MI setting in a nationally representative setting [12]. This focussed on descriptive sequences of disease, but did not incorporate the timing of onset of disease and was limited to in-hospital data only.

Amongst those studies that have sought to ascertain longitudinal multimorbidity trajectories more broadly, a number of approaches have been used [13]. These include multilevel models that incorporate repeated individual-level multimorbidity measures [14–18], multistate transition models [19–22], data mining methods (including Bayesian approaches [12, 23, 24] and pairwise comparison of temporal sequences [25–27]), longitudinal finite mixture modelling (latent class and growth analysis [28–30]) and visual approaches [31, 32]. Many of these approaches are limited to the inclusion of a small number of disease states, do not fully account for the timing of diagnoses and depend on qualitative interpretation of findings. Non-negative matrix factorisation (NMF) presents a means to reduce a large number of temporally sequenced conditions into a smaller number of meaningful and interpretable trajectories [33]. NMF has previously been used to identify multi-disease patterns in population-level, longitudinal electronic health record (EHR) data, whilst accounting for the timing of individual diagnoses [34, 35]. However, NMF has not been applied in a robust, accessible and meaningful epidemiological framework to study the nature and impact of clinical trajectories over time, with a specific disease-focus.

More broadly, mapping multimorbidity clusters enables (i) identification of new mechanisms of disease, (ii) targeted treatment of patient subgroups and (iii) reconfiguration of services to better meet patient needs [36]. In the context of this study, clustering offers the opportunity to discover distinct patient subgroups that experience different clinical trajectories and identify those that are at greatest risk of adverse outcomes amongst the heterogeneous population of individuals presenting with MI [6]. The identification of differential outcomes resulting from such trajectories may inform the future application of targeted interventions, such as enhanced follow-up and personalised risk factor reduction programmes that better meet the needs of specific patient groups.

In this study, we developed an accessible, efficient and robust NMF-based epidemiological framework in order to derive longitudinal multimorbidity trajectories in a nationwide cohort of patients with MI. In doing so, we provide (i) quantification of post-MI acute and chronic disease accrual patterns as observed in a real-world patient population, (ii) characterisation of determinants of distinct post-MI multimorbidity trajectories and (iii) evaluation of the impact of the clusters over time on long-term all-cause mortality.

## Methods

### Data sources

This study used the Wales Multimorbidity e-Cohort (WMC), derived from the Secure Anonymised Information Linkage (SAIL) databank [37, 38]. SAIL includes pseudonymised, linked and routinely collected demographic, health, environmental, social and mortality data for the resident population of Wales. Diagnosis data were available from both primary care (Welsh Longitudinal General Practice [WLGP] data; supplied by ~ 86% of GP practices in Wales) and secondary care (Patient Episode Database for Wales [PEDW]; all inpatient and day case activity undertaken in Wales, plus data on Welsh residents treated in England). Primary care diagnoses were recorded using Read codes; secondary care diagnoses were recorded using the International Classification of Diseases version 10 (ICD-10). The CALIBER phenotyping algorithm was used to derive 306 discrete clinical phenotypes from primary and secondary care diagnostic codes (full list presented in Additional file 1: Table S1) [37, 39, 40]. These were divided into 62 acute conditions and 227 chronic (long-term) conditions. The remainder (*n* = 17) pertained to conditions of childhood and were excluded. All-cause mortality data were made available by the Office for National Statistics, including those that died outside of Wales.

### Study population

The WMC included all Welsh residents aged between 0 and 99 years on 1 January 2000, with detailed diagnostic data accrued only from the cohort inception date (1 January 2000). Our analytical cohort included all individuals with an index MI from 1 January 2005 to 31 December 2019, allowing for a 5-year diagnosis accrual period. MI was defined as per CALIBER [39]. Chronic conditions diagnosed prior to MI were ascertained using all available pre-MI data for each individual, with an unrestricted look-back period. Follow-up time commenced on the date of index MI and continued until censoring (due to death, a break in Welsh residency or the end of the study period [31 December 2019]). Those with MI prior to the study period were excluded. In order to prevent underestimation of diagnoses commonly made in primary care, the present study was limited to the subpopulation of the WMC that were registered at a GP practice contributing to SAIL.

### Non-negative matrix factorisation (NMF)

**Fig. 1:**
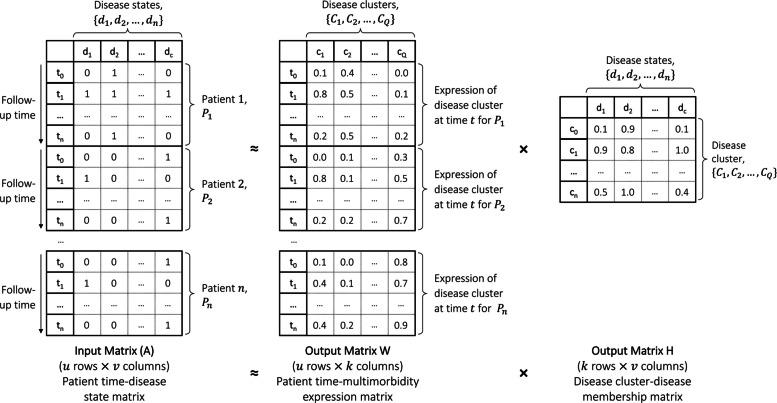
Schematic of the formation and decomposition of the person-time disease matrix used in the present study. Note that the follow-up time for each individual (denoted by tn) extends from the index date (date of MI) to the date of death or censorship and therefore will differ between individuals

Clusters of post-MI multimorbidity over time were ascertained using non-negative matrix factorisation (NMF). To implement NMF, we generated a disease matrix containing an indicator variable for each of the clinical phenotypes for every year of follow-up year for each individual in the analytic cohort, beginning with the date of MI (Fig. 1) [34]. Each condition was modelled either as an acute condition (those with an expected duration of ≤ 1 year, e.g. acute infections) or a chronic condition (those with an expected duration of > 1 year, e.g. heart failure, chronic obstructive pulmonary disease; Additional file 1: Table S1). Acute conditions were indicated in the matrix only for the period of presentation, whereas chronic conditions were indicated for the period of presentation and all successive periods, until censoring or death. Chronic conditions diagnosed in the post-MI period were included from their date of diagnosis; acute events prior to the index MI were not included in post-MI matrices. Concatenation of these matrices for every individual gave the NMF input matrix.

NMF was implemented with simple multiplicative update rules [33, 41] using sparse matrix representations via the *Scikit-learn* package in Python [42]. NMF decomposed the input matrix (matrix $A$; made up of $u$ rows $\times$ $v$ columns) into two matrices made up of non-negative numbers: (i) matrix $W$ ($u$ rows $\times$ $k$ columns), representing the ‘expression’ of each of $k$ *disease clusters* for an individual in each discrete time period, and (ii) matrix $H$ ($k$ rows $\times$ $v$ columns), representing the membership of individual conditions in each disease cluster. The degree of membership of an individual to each of the $k$ *disease clusters* over time is described in this paper as a *multimorbidity trajectory*.

Unlike regression models, NMF does not have a deterministic, ‘best-fit’ solution: matrices $W$ and $H$ were initialised using random values and updated using an iterative process, minimising an appropriate loss function. One hundred repetitions of NMF were performed for the selected value of $k$ to encourage convergence to an optimal solution (preventing local minima due to the random seeding of starting values). In order to determine the optimal number of disease clusters to fit our data, we plotted the mean squared error and Kullback–Leibler divergence (as measures of the model ‘fit’) and cophenetic correlation coefficient (as a measure of model ‘stability’) for 3 to 25 trajectories. The solutions were also reviewed for their clinical interpretability. The final number of disease clusters was chosen to offer the best trade-off between fit, complexity, stability and clinical interpretability.

Our final NMF output produces two key concepts for use in further analyses: these are (i) an individual’s single-year *disease cluster* membership (matrix $W$), which is interpreted as the extent to which an individual belongs to each disease cluster during each year of follow-up (from MI to death or censoring), and (ii) an individual’s predominant *post-MI multimorbidity trajectory*, which is the disease cluster with the greatest area under the cluster-time curve during the follow-up period (from MI to death or censoring).

For additional details of the mathematical basis of these NMF methods, see Additional file 2: Supplementary methods [43, 44].

### Validation of trajectories

Internal validation of cluster stability was performed using non-parametric bootstrapping [45]. Briefly, 1000 bootstrap samples equal in size to the final analytic cohort were drawn (with replacement) from the analytic cohort. The full NMF procedure was applied to each of these bootstrap samples, using the optimal value of $k$. The final trajectory for each individual was identified. Each bootstrap sample may give a potentially different set of trajectory assignments, as the sample composition differs, enabling assessment of how sensitive the NMF algorithm is to changes in the data. Congruent clusters were matched between the original and bootstrap samples [45]. The mean Jaccard index (JI; with corresponding 95% confidence intervals, CI) for each matched cluster was calculated across all bootstrap iterations. A JI of > 0.75 was interpreted to represent a high degree of stability; a JI of < 0.5 suggested an unstable cluster. Intermediate values would suggest NMF is capturing a true pattern in the data, with some uncertainty in between-cluster allocation.

### Statistical analysis

Patient demographics, including age, sex, year of MI diagnosis, socioeconomic deprivation (measured using the Welsh Index of Multiple Deprivation [WIMD] [46]) and rural–urban status, were summarised for the analytic cohort and stratified for patients in each post-MI multimorbidity trajectory. Multinomial logistic regression was used to identify sociodemographic factors associated with each cluster. Restricted mean survival time (RMST) was calculated for patients following each multimorbidity trajectory using the Kaplan–Meier method. Disease clusters and multimorbidity trajectories were labelled in ascending numerical order by their associated RMST. Royston-Parmar flexible parametric survival modelling was used to evaluate the association of each multimorbidity trajectory with all-cause mortality. Fully adjusted hazard ratios (HR) and 95% CI accounting for age, sex, year of MI, WIMD and rural–urban status were reported alongside unadjusted HRs and 95% CIs. Throughout the follow-up period, the dominant post-MI disease cluster was modelled as a categorical, time-updating covariate. Survival was modelled on the log cumulative hazard scale, with 6 degrees of freedom (as determined through identification of the minima of the Bayesian Information Criterion [BIC] and visual inspection of how well the model captured the Nelson-Aalen cumulative hazard function). Non-linearity of age was modelled using restricted cubic spline functions with three degrees of freedom. Unadjusted Kaplan–Meier and model-derived, adjusted standardised survival curves were plotted [47]. The inclusion of covariates in the model was guided by a priori identification of potential confounders of the exposure and outcome association. All survival modelling was performed using *stpm3* and *standsurv* (Stata MP, version 18).

Due to the robust nature of data linkage in SAIL and the restriction of this study to those individuals included in the WMC e-Cohort and registered with a SAIL-contributing GP practice, there were no missing data for the covariates included in this analysis.

### Ethics, data governance and reporting

The use of de-identified data in this project was approved by the SAIL Information Governance Review Panel (26 June 2019, project 0911). The SAIL databank has ongoing ethical approval from the UK National Research Ethics Service (NRES) [48]. All data were stored and analysed in an ISO27001 and UK Statistics Authority (UKSA) Digital Economy Act (DEA) accredited Trusted Research Environment (TRE). This study is reported in accordance with the STrengthening the Reporting of OBservational studies in Epidemiology (STROBE) statement [49], REporting of studies Conducted using Observational Routinely-collected Data (RECORD) guideline [50] and the CODE-EHR framework [51] (Additional file 3 and Additional file 4). Data used for this study are available via application to the SAIL Databank.

### Role of the funding source

This project was funded by the AI for Science and Government Fund, via the Alan Turing Institute Health Programme (TU/ASG/R-SPEH-114). JB received funding from the Wellcome Trust 4ward North Clinical Research Training Fellowship (ref: 227,498/Z/23/Z; R127002). CH was funded by a British Heart Foundation-Turing Cardiovascular Data Science Award (BHF-Turing-19/02/1022). MH was funded by the Wellcome Trust (ref: 206470/Z/17/Z). The funders had no role in the study design, collection, analyses or interpretation of data or in the writing of the report or decision to submit the paper for publication.

## Results

**Fig. 2:**
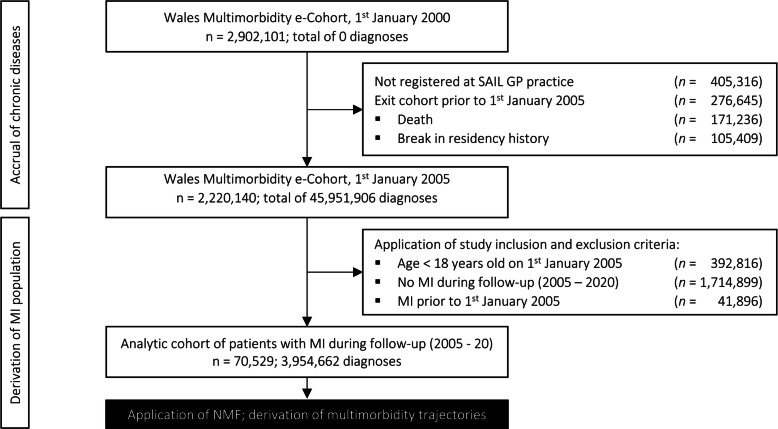
The flow of participants from the WMC e-Cohort to the final analytic cohort

In total, *n* = 2,902,101 individuals entered the Wales Multimorbidity Cohort on 1 January, 2000 (> 99% of the total population of Wales; Fig. 2). Of these, *n* = 2,220,140 remained under follow-up on 1 January 2005. Following the application of the study in- and exclusion criteria, 70,529 individuals with an index MI between 1 January 2005 and 31 December 2019 were included in the analytic cohort, with a total of 308,269 person-years of follow-up. The median age at MI was 72 (interquartile range: 62–82) years and 28,623 (40.6%) were female (Table 1). There was a higher proportion of individuals from the most deprived areas of Wales (top quintile; 23.7%) compared with those in the least deprived areas (second to fifth quintiles; 21.4, 20.2, 17.6 and 17.1%, respectively). The overall restricted mean survival time following MI was 8.1 years (95% confidence intervals 8.0–8.1 years).

**Table 1:** Baseline characteristics of the patients included in this study, in the whole analytic cohort and stratified by post-MI multimorbidity trajectory

	All patients with MI (*n* = 70,529)	Predominant post-MI multimorbidity trajectory									
		T_1_	T_2_	T_3_	T_4_	T_5_	T_6_	T_7_	T_8_	T_9_	T_10_
		Mental health conditions and obesity	Angina and hypertension	Musculoskeletal conditions	Nonspecific conditions	Anaemia and GI conditions	Diabetes and related conditions	Lung disease	HF, AF and valvular disease	Dementia, stroke and AF	Recurrent MI and acute infection
		(*n* = 4803)	(*n* = 8468)	(*n* = 4710)	(*n* = 3660)	(*n* = 3469)	(*n* = 5544)	(*n* = 3063)	(*n* = 6135)	(*n* = 4642)	(*n* = 26,035)
Age at MI—y, median (IQR)	72 (62–82)	57 (49–65)	64 (55–73)	66 (57–75)	73 (64–81)	71 (62–79)	67 (58–76)	71 (63–79)	74 (64–84)	78 (70–84)	79 (70–85)
Female, *n* (%)	28,623 (40.6)	1655 (34.5)	2385 (28.2)	2192 (46.5)	1085 (30.0)	1421 (41.0)	1732 (31.2)	1472 (48.1)	2304 (37.6)	2302 (49.6)	12,075 (46.4)
Welsh IMD quintile, *n* (%)											
1—most deprived	16,742 (23.7)	1435 (29.9)	1832 (21.6)	1089 (23.1)	791 (21.6)	826 (23.8)	1442 (26.0)	823 (26.9)	1310 (21.4)	974 (21.0)	6308 (24.2)
2	15,077 (21.4)	1089 (22.7)	1759 (20.8)	1007 (21.4)	783 (21.4)	731 (21.1)	1260 (22.7)	670 (21.9)	1271 (20.7)	955 (20.6)	5581 (21.4)
3	14,250 (20.2)	904 (18.8)	1730 (20.4)	948 (20.1)	727 (19.9)	698 (20.1)	1082 (19.5)	620 (20.2)	1222 (19.9)	951 (20.5)	5273 (20.3)
4	12,386 (17.6)	704 (14.7)	1576 (18.6)	840 (17.8)	710 (19.4)	611 (17.6)	885 (16.0)	510 (16.7)	1196 (19.5)	946 (20.4)	4499 (17.3)
5—least deprived	12,074 (17.1)	671 (14.0)	1571 (18.6)	826 (17.5)	649 (17.7)	603 (17.4)	875 (15.8)	440 (14.4)	1136 (18.5)	816 (17.6)	4374 (16.8)
Rural–urban status, *n* (%)											
Urban (> 10,000)	48,039 (68.1)	3437 (71.6)	5650 (66.7)	3167 (67.2)	2504 (68.4)	2313 (66.7)	3806 (68.7)	2117 (69.1)	4034 (65.8)	3139 (67.6)	17,872 (68.6)
Town and fringe	12,450 (17.7)	849 (17.7)	1457 (17.2)	863 (18.3)	630 (17.2)	655 (18.9)	971 (17.5)	517 (16.9)	1098 (17.9)	786 (16.9)	4624 (17.8)
Rural	10,040 (14.2)	517 (10.8)	1361 (16.1)	680 (14.4)	526 (14.4)	501 (14.4)	767 (13.8)	429 (14.0)	1003 (16.3)	717 (15.4)	3539 (13.6)
Selected LTCs prior to MI, *n* (%)											
Hypertension	32,544 (46.1)	1557 (32.4)	3563 (42.1)	2004 (42.5)	1440 (39.3)	1476 (42.5)	3057 (55.1)	1402 (45.8)	3075 (50.1)	2321 (50.0)	12,649 (48.6)
Diabetes mellitus	20,445 (29.0)	936 (19.5)	1277 (15.1)	905 (19.2)	824 (22.5)	849 (24.5)	4092 (73.8)	646 (21.1)	1349 (22.0)	1132 (24.4)	8435 (32.4)
Chronic pulmonary disease	23,100 (32.8)	1346 (28.0)	1615 (19.1)	1289 (27.4)	1814 (49.6)	1116 (32.2)	1391 (25.1)	1614 (52.7)	1518 (24.7)	1263 (27.2)	10,134 (38.9)
Heart failure	10,465 (14.8)	282 (5.9)	349 (4.1)	276 (5.9)	372 (10.2)	346 (10.0)	685 (12.4)	376 (12.3)	1489 (24.3)	631 (13.6)	5659 (21.7)
Peripheral vascular disease	9116 (12.9)	282 (5.9)	487 (5.8)	291 (6.2)	328 (9.0)	370 (10.7)	928 (16.7)	682 (22.3)	672 (11.0)	507 (10.9)	4569 (17.5)
Cancer	15,273 (21.7)	427 (8.9)	1081 (12.8)	711 (15.1)	752 (20.5)	680 (19.6)	825 (14.9)	759 (24.8)	1142 (18.6)	1460 (31.5)	7436 (28.6)
Chronic kidney disease	15,177 (21.5)	375 (7.8)	790 (9.3)	608 (12.9)	613 (16.7)	660 (19.0)	1372 (24.7)	555 (18.1)	1383 (22.5)	961 (20.7)	7860 (30.2)
Chronic liver disease	973 (1.4)	214 (4.5)	66 (0.8)	35 (0.7)	36 (1.0)	50 (1.4)	74 (1.3)	34 (1.1)	46 (0.7)	36 (0.8)	382 (1.5)
Connective tissue disease	6125 (8.7)	231 (4.8)	383 (4.5)	779 (16.5)	251 (6.9)	273 (7.9)	338 (6.1)	259 (8.5)	409 (6.7)	376 (8.1)	2826 (10.9)
Dementia	3054 (4.3)	50 (1.0)	76 (0.9)	64 (1.4)	56 (1.5)	59 (1.7)	73 (1.3)	51 (1.7)	119 (1.9)	714 (15.4)	1792 (6.9)
Obesity	6894 (9.8)	679 (14.1)	597 (7.1)	452 (9.6)	292 (8.0)	327 (9.4)	1027 (18.5)	226 (7.4)	550 (9.0)	305 (6.6)	2439 (9.4)
Number of LTCs prior to MI, median (IQR)	8 (5–12)	6 (4–10)	5 (3–8)	7 (4–11)	8 (5–12)	8 (5–12)	8 (5–12)	8 (5–12)	8 (5–11)	8 (5–12)	9 (6–14)
RMST*—y (95% CI)	8.1 (8.0–8.1)	13.1 (12.9–13.2)	12.8 (12.6–12.9)	12.7 (12.6–12.8)	10.9 (10.7–11.1)	10.8 (10.6–11.0)	10.2 (10.0–10.4)	8.6 (8.4–8.8)	8.1 (7.9–8.4)	7.7 (7.5–7.9)	2.9 (2.8–3.0)

*Abbreviations*: *IMD* index of multiple deprivation, *LTCs* long-term conditions, *RMST* restricted mean survival time, *SD* standard deviation, *WMC* Wales Multimorbidity Cohort, *y* years

^*^Restricted mean survival time is calculated as per the Kaplan–Meier method, with continuity correction

### Accrual of multimorbidity

At the time of index MI, *n* = 67,023 (95.0%) patients had ≥ 2 long-term conditions (of a total of 227 chronic conditions), with a median (interquartile range, IQR) of 8 (5–12) long-term conditions. The most common pre-existing long-term conditions included hypertension (46.1%), chronic pulmonary disease (32.8%), diabetes mellitus (29.0%), cancer (21.7%) and chronic kidney disease (21.5%). At 1 year post-MI, 50,633 of 50,737 surviving patients (99.8%) had ≥ 2 long-term conditions (median [IQR] = 12 [7–17]). In the post-MI period, the most common new long-term diagnoses were angina (14,848; 21.1%), heart failure (10,915; 15.5%), hypertension (8788; 12.5%), anaemia (8050; 11.4%), atrial fibrillation (7645; 10.8%), cataract (6309; 8.9%), osteoarthritis (5498; 7.8%), COPD (5022; 7.1%), diabetes mellitus (4943; 7.0%) and gastro-oesophageal reflux disease (4644; 6.6%). The most frequent acute diagnoses occurring once or more in the post-MI period were infections: unspecified (21,010; 29.8%), lower respiratory tract (17,325; 24.6%) and urinary (10,297; 14.6%), and acute kidney injury (10,577; 15.0%).

### Disease clusters and multimorbidity trajectories

**Fig. 3:**
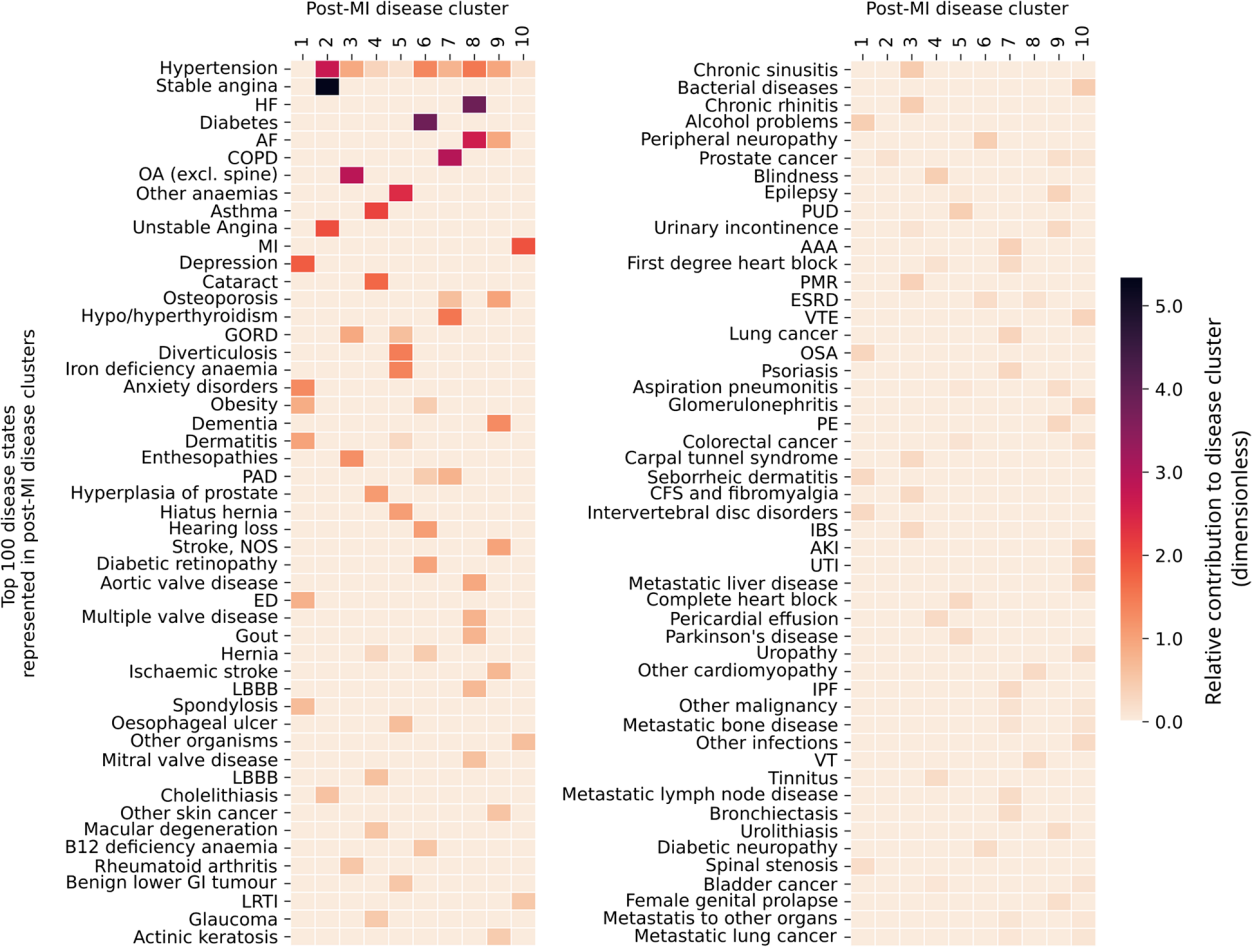
The top 100 disease states represented in one or more post-MI disease cluster. Diseases are shown in descending order of total contribution to one or more clusters. The remaining 136 conditions included in this study also contribute to trajectories but to a lesser extent and are not depicted. This is a graphical representation of *matrix H*. Abbreviations: AAA abdominal aortic aneurysm, AKI acute kidney injury, COPD chronic obstructive pulmonary disease, ED erectile dysfunction, ESRD end-stage renal disease, GORD gastrointestinal reflux disease, HF heart failure, IBS irritable bowel syndrome, IPF idiopathic pulmonary fibrosis, LBBB left bundle branch block, LRTI lower respiratory tract infection, MD macular degeneration, MI (recurrent) myocardial infarction, NOS not otherwise specified, OA osteoarthritis, OSA obstructive sleep apnoea, PAD peripheral arterial disease, PE pulmonary embolism, PMR polymyalgia rheumatica, PUD peptic ulcer disease, RA rheumatoid arthritis, RBBB right bundle branch block, UTI urinary tract infection, VHD other valvular heart disease, VTE venous thromboembolism, VT ventricular tachycardia

The optimal NMF solution, which minimised model fit statistics (Kullback–Leibler divergence and residual sum of squares) and displayed good clinical interpretability, resulted in ten post-MI disease clusters (C_1_ to C_10_; Fig. 3; Additional file 5: Fig. S1; Additional file 6: Table S2). The corresponding multimorbidity trajectories (T_1_ to T_10_) represent the most frequently observed disease cluster during follow-up for that individual.

Descriptive clinical labels were applied to each cluster, with the same descriptor used for each corresponding multimorbidity trajectory, as follows: C_1_ ‘mental health and obesity’ characterised by a combination of mental health conditions (including anxiety, depression and alcohol-associated disorders) and obesity-related conditions (including obesity, obstructive sleep apnoea and intervertebral disc disorders); C_2_ ‘angina and hypertension’ including recurrent presentation with angina (both stable and unstable) and hypertension; C_3_ ‘musculoskeletal conditions’ including arthritis and inflammatory-related conditions (osteoarthritis, enthesopathies and synovial disorders); C_4_ ‘nonspecific’ lacked a congruent theme, but included respiratory, ophthalmological and other conditions; C_5_ ‘anaemia and gastrointestinal conditions’ was characterised by haemorrhagic gastrointestinal conditions (including diverticular disease, oesophagitis and oesophageal ulceration, gastro-oesophageal reflux, colonic polyps and peptic ulcer disease) and anaemia; C_6_ ‘diabetes and related conditions’ characterised by metabolic conditions, including obesity, diabetes mellitus and cardiometabolic complications such as diabetic retinopathy, peripheral neuropathy and peripheral arterial disease; C_7_ ‘lung disease’ including chronic obstructive pulmonary disease, thyroid dysfunction as well as peripheral arterial disease and aortic abdominal aneurysm; C_8_ ‘heart failure, arrhythmia and valvular heart disease’ including heart failure, hypertension, valvular heart disease and atrial fibrillation; C_9_ ‘dementia, stroke and atrial fibrillation’ included causes and consequences of cerebrovascular and cerebroembolic disease; C_10_ ‘recurrent MI and acute infection’ included recurrent MI and lower respiratory tract infection, urinary tract infection and bacterial infections (Table 2).

**Table 2:** The conditions with the greatest weight in each disease cluster, listed in decreasing order of the importance of each condition to each cluster

Cluster	Top 5 conditions		Other notable conditions	Narrative description
C_1_	1	Depression	Spondylosis, alcohol problems, obstructive sleep apnoea, seborrheic dermatitis, intervertebral disc disorders	Mental health and obesity-related conditions
	2	Anxiety		
	3	Dermatitis		
	4	Obesity		
	5	Erectile dysfunction		
C_2_	1	Stable angina	N/A	Angina and hypertension
	2	Hypertension		
	3	Unstable angina		
	4	Cholelithiasis		
	5	Primary malignancy of prostate		
C_3_	1	Osteoarthritis	Chronic sinusitis, allergic and chronic rhinitis, polymyalgia rheumatica, carpal tunnel syndrome, postviral fatigue syndrome, neurasthenia and fibromyalgia	Musculoskeletal conditions
	2	Enthesopathies and synovial disorders		
	3	Hypertension		
	4	Gastro-oesophageal reflux disease		
	5	Rheumatoid arthritis		
C_4_	1	Asthma	Glaucoma, visual impairment and blindness, hypertension, abdominal hernia	Nonspecific conditions (including asthma and eye conditions)
	2	Cataract		
	3	Hyperplasia of prostate		
	4	Right bundle branch block		
	5	Macular degeneration		
C_5_	1	Other anaemia	Gastro-oesophageal reflux disease, benign neoplasm of colon, rectum and anal canal, peptic ulcer disease, dermatitis	Anaemia and gastrointestinal conditions
	2	Diverticular disease of the intestine		
	3	Iron deficiency anaemia		
	4	Diaphragmatic hernia		
	5	Oesophagitis and oesophageal ulcer		
C_6_	1	Diabetes mellitus	Peripheral arterial disease, abdominal hernia, obesity	Diabetes mellitus and related conditions
	2	Hypertension		
	3	Hearing loss		
	4	Diabetic ophthalmic complications		
	5	Vitamin B12 deficiency anaemia		
C_7_	1	COPD	Aortic abdominal aneurysm, primary malignancy of the lung and trachea, psoriasis, interstitial pulmonary disease	Lung disease
	2	Hypo- or hyperthyroidism		
	3	Peripheral arterial disease		
	4	Hypertension		
	5	Osteoporosis		
C_8_	1	Heart failure	Gout, left bundle branch block, nonrheumatic mitral valve disorders, other cardiomyopathy, ventricular tachycardia	HF, AF, valvular heart disease and hypertension
	2	Atrial fibrillation		
	3	Hypertension		
	4	Non-rheumatic aortic valve disorders		
	5	Multiple valve disease		
C_9_	1	Dementia	Ischaemic stroke, epilepsy, pulmonary embolism, actinic keratosis, primary malignancy of skin and other subcutaneous tissue	Dementia, stroke and AF
	2	Stroke not otherwise specified		
	3	Osteoporosis		
	4	Hypertension		
	5	Atrial fibrillation		
C_10_	1	Recurrent myocardial infarction	Glomerulonephritis, acute kidney injury, urinary tract infections, obstructive and reflux uropathy	Recurrent MI and acute infections
	2	Other or unspecified infectious organisms		
	3	Lower respiratory tract infection		
	4	Bacterial diseases (excluding TB)		
	5	Venous thromboembolic disease		

*Abbreviations*: *AF* atrial fibrillation, *COPD* chronic obstructive pulmonary disease, *MI* myocardial infarction, *N/A* not applicable, *TB* tuberculosis. Note: the choice of narrative description is also informed by the intensity by which a condition is represented in a trajectory (i.e. the darker a colour box on the heatmap in Fig. 3, the more strongly it is represented in the trajectory)

Cluster stability was evaluated using bootstrapping (1000 replicates) and the Jaccard index. The identified trajectories were reproducibly identified across the bootstrapped replicates, with mean trajectory-wise Jaccard indices ranging from 0.70 (95% CI 0.61–0.79; T_4_) to 0.95 (95% CI 0.86–1.00; T_10_) indicating moderate-to-high stability across all identified trajectories. The lower bounds of the 95% CI were above 0.5 for all trajectories, suggesting that this clustering solution is robust to sampling variability.

### Characteristics of patients by multimorbidity trajectory

T_1_ (‘mental health and obesity’) included individuals that were younger at MI (median 57 vs. 72 years; *p* < 0.001), fewer women (34.5 vs. 40.6%; *p* < 0.001) and had a lesser burden of pre-existing multimorbidity (median [IQR] 6 [4−10] LTCs; Table 1). T_10_ (‘recurrent MI and acute infection’) was observed in individuals that were older at the time of MI (median 79 vs. overall median of 72 years; *p* < 0.001), included a greater proportion of women (46.4 vs. 40.6%; *p* < 0.001), and those that had a greater burden of multimorbidity prior to MI (median [IQR] of 9 [6–14] LTCs). The age at MI, age in a given period of follow-up and the sex of the individual were the strongest determinant of an individual’s trajectory membership in a given year following MI (Additional file 7: Table S3).

### All-cause mortality by trajectory

**Fig. 4:**
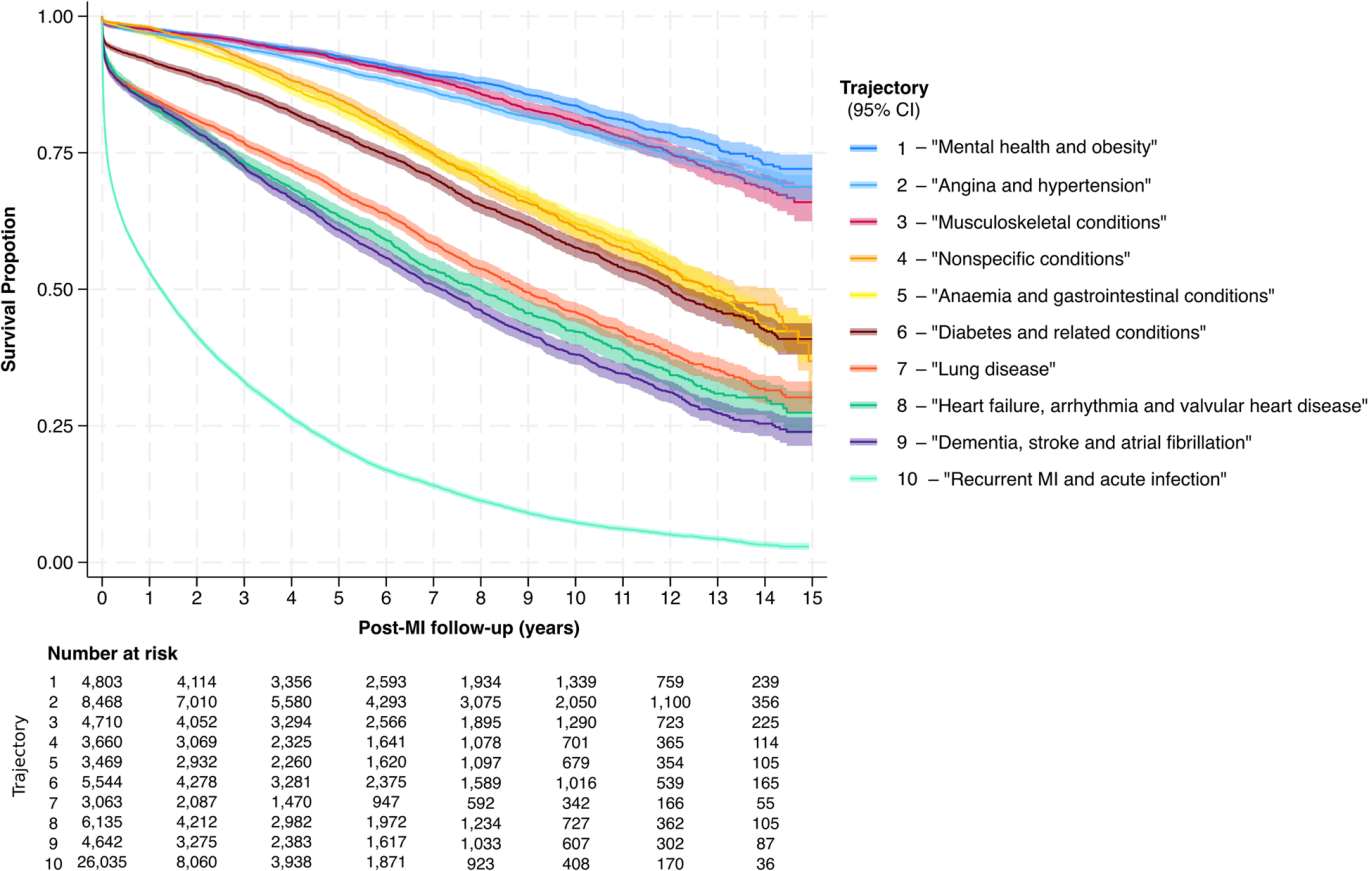
Unadjusted post-MI Kaplan–Meier survival, by single most predominant trajectory during follow-up. The shaded areas represent 95% confidence intervals

**Fig. 5:**
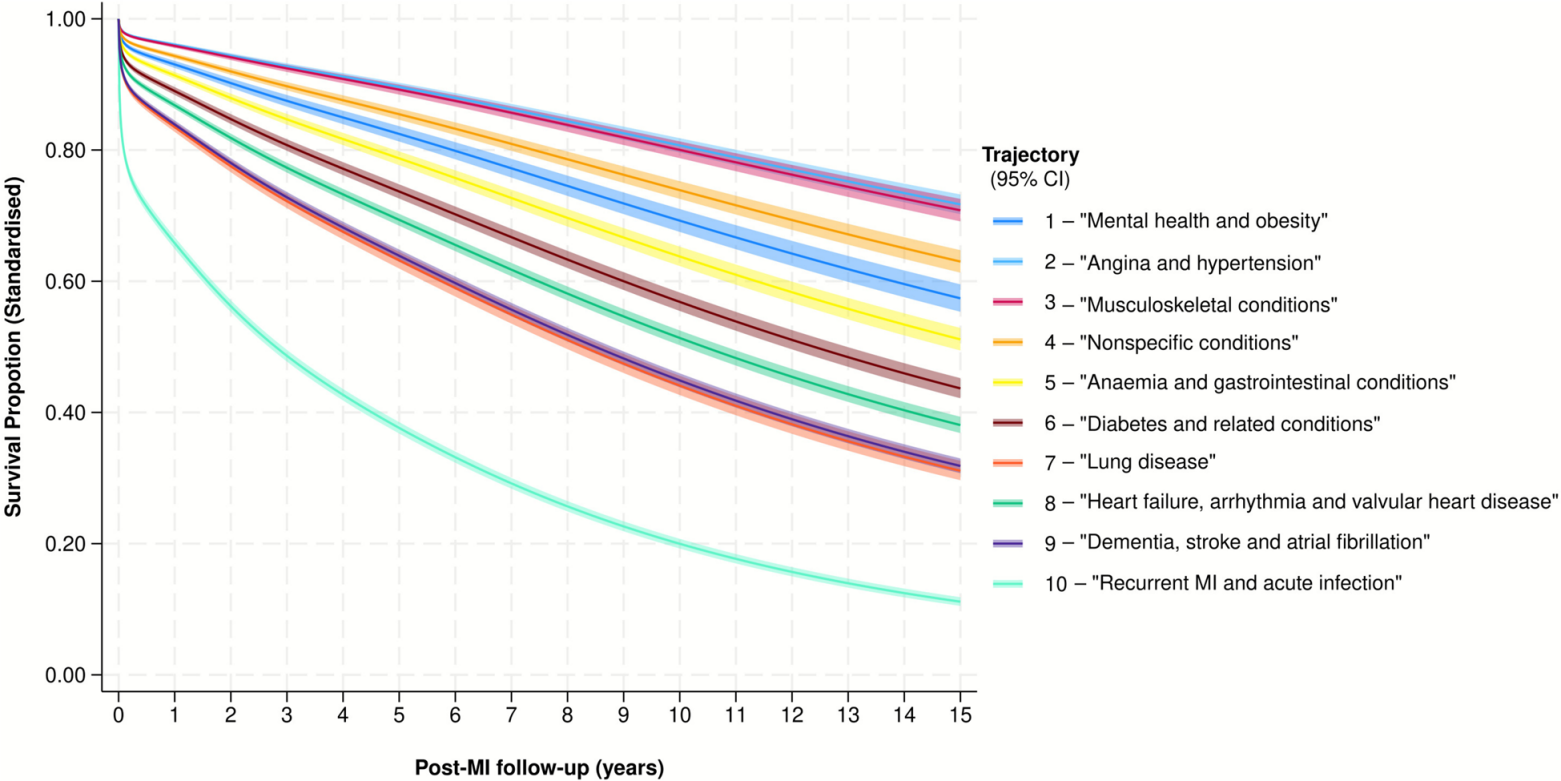
Standardised survival curves for each trajectory, adjusted for age, sex, year of MI, socioeconomic deprivation and rurality. The shaded areas represent 95% confidence intervals

Crude survival over the follow-up period was highest for T_1_ ‘mental health and obesity’, T_2_ ‘angina and hypertension’ and T_3_ ‘musculoskeletal conditions’ and significantly poorer for T_10_, which had very high early post-MI mortality (Fig. 4). Restricted mean survival time ranged from 13.1 (95% CI 12.9–13.2) years in T_1_ (‘mental health and obesity’) to 2.9 (95% CI 2.8–3.0) years in T_10_ (‘recurrent MI and acute infection’; Table 1). After adjustment for age, sex, year of MI, socioeconomic deprivation and rurality, there was a significant difference in survival for each trajectory relative to the baseline trajectory (T_1_), with T_10_ associated with the greatest risk of mortality (hazard ratio [HR] 6.62; 95% confidence interval [CI]: 6.09–7.20) and T_2_ ‘angina and hypertension’ associated with the lowest adjusted risk of mortality (HR 0.55, 95% CI: 0.50–0.61) relative to T_1_ (Table 3, Fig. 5).

**Table 3:** The association between each post-MI multimorbidity trajectory and all-cause mortality

Trajectory	HR (95% CI) for association between post-MI multimorbidity trajectory and long-term all-cause survival	
	Unadjusted (crude)	Fully adjusted^†^
T_1_—‘mental health and obesity’ (*n* = 4803; total: 31,736 py)	Ref*	Ref*
T_2_—‘angina and hypertension’ (*n* = 8468; total: 53,001 py)	1.23 (1.12–1.36)	0.55 (0.50–0.61)
T_3_—‘musculoskeletal conditions’ (*n* = 4710; total: 33,892 py)	1.16 (1.04–1.30)	0.57 (0.52–0.64)
T_4_—‘nonspecific conditions’ (*n* = 3660; total: 26,503 py)	2.34 (2.11–2.58)	0.80 (0.73–0.89)
T_5_—‘anaemia and GI conditions’ (*n* = 3469; total: 21,025 py)	2.42 (2.19–2.68)	1.26 (1.14–1.39)
T_6_—‘diabetes and related conditions’ (*n* = 5544; total: 27,724 py)	2.93 (2.67–3.21)	1.65 (1.50–1.81)
T_7_—‘lung disease’ (*n* = 3063; total: 13,015 py)	4.94 (4.49–5.44)	2.61 (2.38–2.86)
T_8_—‘HF, arrhythmia and valvular disease’ (*n* = 6135; total: 27,044 py)	4.39 (4.02–4.79)	2.01 (1.84–2.20)
T_9_—‘dementia, stroke and AF’ (*n* = 4642; total: 22,098 py)	5.38 (4.93–5.88)	2.53 (2.33–2.76)
T_10_—‘recurrent MI and acute infection’ (*n* = 26,035; total: 52,231 py)	17.21 (15.88–18.66)	6.62 (6.09–7.20)

All-cause mortality was modelled using Royston-Parmar flexible parametric survival models (log cumulative hazard scale; 6 degrees of freedom). *n* represents the number of patients for which the listed trajectory was observed for the longest proportion of follow-up; the second number represents the total number of person-years with that trajectory during follow-up

*Abbreviations*: *AF* atrial fibrillation, *CI* confidence interval, *GI* gastrointestinal, *HF* heart failure, *HR* hazard ratio, *py* person-years, *Ref* reference category, *WIMD* Wales Index of Multiple Deprivation

^*^T_1_ was used as the referent category as this was associated with the greatest absolute crude survival over the course of follow-up

^†^Adjustment was performed for age, sex, year of MI, WIMD and rurality, modelling trajectory as a time-varying covariate and allowing non-proportional effects. Adjustment for age was performed by introducing a restricted cubic spline term (5 degrees of freedom) for the age of a person at the time of their MI. Adjustment for WIMD and rural–urban status was performed by introducing indicator variables, as per the categories displayed in Table 1

## Discussion

### Key findings

In this study, we identified 10 distinct multimorbidity trajectories following MI for 70,529 individuals in Wales, between 2005 and 2020. These trajectories were associated with substantially different survival rates that persisted following adjustment for patient demographics including age, sex, year of MI, socioeconomic deprivation and rurality. The most adverse trajectory, associated with high risk of early all-cause mortality post-MI (T_10_; ‘recurrent MI and acute infection’; followed by 36.9% of the cohort), was characterised by recurrent MI, acute infections and renal conditions and was observed more commonly in men, older individuals and those that had a greater burden of baseline comorbidity. In contrast, the trajectory with the lowest risk of all-cause mortality included a combination of mental health conditions (including anxiety, depression and alcohol-associated disorders) and obesity-related conditions (including obesity, obstructive sleep apnoea and intervertebral disc disorders; T_1_, ‘mental health and obesity’; followed by 6.8% in the cohort) which was more commonly observed amongst female patients and those that were younger at time of MI diagnosis. The distinct nature of these trajectories and their differential outcomes clearly highlights that a ‘one-size fits all’ programme of post-MI management is insufficient, and a tailored programme of follow-up is likely highly beneficial.

### Methodological adaptations and their implications

We adapted previously reported NMF-based methodologies [34] in a number of important ways. Firstly, the timescale on which NMF was applied was post-MI patient-years (1-year intervals from the date of MI), as opposed to discrete years of patient age. Secondly, previous approaches weighted conditions by their inverse prevalence in a population (‘disease frequency, inverse patient frequency’). This assigned disproportionate weighting to infrequently occurring conditions within clusters. As common diseases may be as impactful as rare diseases to an individual, we weighted all conditions equally. Equal weighting avoids over-emphasising either very common or very rare conditions. If occurrence (frequency) weighting were used, clusters would be driven mainly by highly prevalent chronic conditions (e.g. hypertension and osteoarthritis), likely diluting signals from acute events (e.g. infection, AKI) and reducing clinical specificity of the trajectories. Inverse-frequency weighting (as reported in prior studies [34]) would accentuate rare diagnoses in trajectories over those with an overall greater clinical burden. An equal weighting strategy was therefore used to prevent either very common or very rare diagnoses disproportionately driving clusters. This strategy may have reduced comparability to prior NMF studies, but increased comparability to prior epidemiological studies that evaluated multimorbidity in patients with MI, as supported by Patient and Public Involvement and Engagement (PPIE) conducted alongside this project.

Finally, previous studies excluded acute conditions from analyses or treated them the same as chronic conditions. Given recent recommendations to include both acute and chronic conditions in multimorbidity research [52], we modelled the occurrence of both acute and chronic conditions, whilst also accounting for the different nature of acute vs. chronic conditions directly with respect to their occurrence within a disease cluster.

### Integration with wider literature

Whilst direct external validation of our findings in other patient cohorts with comparable linked data at a full population scale was precluded due to strict governance processes and associated barriers to timely access to EHR data, we were instead able to compare our findings to a number of recent studies in other national populations—albeit in studies which did not incorporate the time of onset of post-MI disease accrual and multi-disease interactions. Adverse trajectories including recurrent ischaemic events and recurrent cardiovascular events, similar to those observed in our most adverse trajectory (T_10_), were also observed in our previous descriptive work of the sequential accrual of conditions following MI amongst 145 million hospitalisation records in England [12] and amongst 6.2 million individuals in a study of the Danish population [27]. Given that trajectories that include recurrent cardiovascular events were observed despite the widespread use of contemporary, guideline-directed secondary prevention therapies post-MI suggests that additional risk factor modification may be beneficial for patients with such trajectories [53]—especially older males at time of MI in which this trajectory was most commonly observed. Whilst we did not assess the impact of the use of secondary preventative medication directly within this study—intensified secondary prevention of shared modifiable risk factors and enhanced post-MI health surveillance may mitigate against increased potential healthcare usage and premature death for individuals with recurrent cardiovascular events identified in this population.

The mental health and obesity trajectory, which includes a combination of mental health conditions (including anxiety, depression and alcohol-associated disorders) and obesity-related conditions (including obesity, obstructive sleep apnoea and intervertebral disc disorders) in this study, occurred more commonly amongst females with MI and those at a younger age of MI. This finding aligns directly with our previous research of the risk of single health outcomes following MI across the population of England in which we also identified an increased risk of depression following MI amongst individuals who were female and those who were diagnosed at younger age of MI [7]. Whilst our current work was able to provide a much greater insight into the co-occurrence of mental health conditions in combination with obesity-related conditions due to the inclusion of primary care data, these consistent health inequalities observed in two separate nationwide populations clearly support the need for a tailored programme of post-MI management to suit the needs of the specific patient groups which follow these trajectories.

To date, many studies that have sought to evaluate the multimorbidity in the context of MI have been limited to the cross-sectional ascertainment of small numbers of long-term conditions prior to MI diagnosis [6, 11, 54]. Indeed, the largest study to perform clustering in this context to date evaluated a total of 21 long-term conditions [11]. Given the present study included 227 chronic and 62 acute conditions spanning all body systems, it is unsurprising that novel combinations of conditions have been identified within our findings. Whilst validation of these novel clusters in other populations should be investigated in future studies, internal validation via bootstrap perturbations of the study cohort yielded reproducible results providing some assurance that these may represent novel clinical phenotypes that warrant additional clinical attention in the post-MI period.

One trajectory identified in the present study included a number of gastrointestinal conditions in combination with anaemia (T_5_). In part, this observation may be attributed to the gastro-erosive side effects of secondary prevention antiplatelet medications, which may lead to gastro-oesophageal irritation, ulceration and bleeding [55, 56]. There has also been a suggestion that patients receiving dual antiplatelet agents in the post-MI setting may have indolent colorectal cancers ‘unmasked’ at an earlier stage due to (i) dual antiplatelet agents causing greater amounts of tumour-associated bleeding and (ii) a greater awareness of reporting bleeding symptoms amongst this patient group [57]. Patients who are likely to follow this trajectory may derive greatest benefit from the use of gastro-protective medications (such as proton-pump inhibitors), as recommended in recent guidelines [58].

To date, NMF has been applied to longitudinal healthcare data in only two studies [34, 35]. Chen et al. applied NMF to a small electronic health record (EHR) dataset made up of cases and controls, in order to stratify patients into phenotypes that were then used to predict whether a patient would develop heart failure or end-stage renal disease [35]. Subsequently, Hassaine et al. applied NMF on a large, unselected population-level electronic health record data to identify disease clusters, using these to create multimorbidity networks [34]. However, neither of these studies presented results that would be interpretable or otherwise of use to an epidemiological or clinical audience. By incorporating our derived trajectories into survival models as time-updating covariates, we demonstrate how complex, high-dimensional, temporally sequenced EHR data can be incorporated into a robust epidemiological outcomes framework that highlights areas of greatest need which would benefit from a personalised approach to management of the post-MI period. This might include enhanced follow-up programmes and personalised risk factor reduction programmes specific to individuals likely to experience recurrent ischaemic disease (especially amongst older males with MI) compared with screening interventions that could be considered for those at risk of mental health and obesity-related trajectories (especially amongst younger females with MI) or use of proton-pump inhibitors for individuals in the anaemia and gastrointestinal condition trajectory.

### Strengths and limitations

This study characterised the multimorbidity trajectories for all individuals in Wales that had incident MI over a 15-year period, using linked, longitudinal primary and secondary care data. Both acute and chronic conditions contributed to the disease trajectories. The stability of the identified trajectories to changes in the underlying study population was robustly demonstrated using parametric bootstrapping and cluster-wise calculation of the Jaccard index. The output of NMF (the per-year expression of each trajectory for each patient) was incorporated as a time-updating covariate within a flexible parametric survival modelling framework. This enabled each trajectory to have a time-varying effect on all-cause mortality and allowed the association of each trajectory with a clinical outcome (all-cause mortality) to be adjusted for covariates and directly interpreted. Furthermore, a strength of our approach allowed us to incorporate both the long-term presence of chronic conditions and the short-term occurrence of acute conditions for the first time in a trajectory model. Our approach could be further applied within a framework of predictive modelling (for example, the pre-MI trajectory data for an individual over time could be used to identify those at greatest risk of post-MI deterioration and mortality) that leverages all the temporally sequenced diagnosis information captured on an electronic health record. Such an approach would further provide clinical value to predict patients that may benefit from additional follow-up and/or therapies targeted to specific trajectories.

We recognise a number of limitations. Firstly, whilst we have used NMF to model many aspects of the real-world complexity of disease accrual over time and are assured by strong agreement of our key findings with other large scale nationwide studies, we acknowledge that some of the trajectories identified require external validation in other populations before being able to impact upon clinical practice. In addition, further methodological developments are required, including approaches which can directly model transitions between trajectories over time per individual, to facilitate individualised risk prediction. Here we modelled the dominant trajectory per individual in order to maintain interpretability of our findings. The results of performing NMF may vary with alternative feature-weighting schemes (e.g. occurrence or inverse-frequency); we prioritised equal weighting for clinical interpretability but acknowledge reduced comparability with prior inverse-weighted NMF analyses [34].

The limited use of NMF to date as a tool in clinical epidemiology likely stems from perceptions of complex interpretations, restrictive data types, lack of implementation in established software packages and high computational complexity (particularly for large datasets, in common with other clustering approaches) [59]. The factorisation procedure is a non-deterministic, non-convex optimisation problem: each factorisation depends on the set of random values used to populate matrices $W$ and $H$ [60]. As such, multiple runs are required (each with random starting values) in order to obtain a factorisation with the best possible ‘fit’ to the original data. NMF is a parametric algorithm that will factorise a matrix into two smaller matrices of rank $k$. There is no empirical best method to calculate the optimal value of $k$; as such, the analyst must select the value of $k$—similar to other clustering approaches including latent class analyses. We used a number of complementary approaches to converge on an optimal value for $k$, which included the minimisation of a penalised error term, the maximisation of the cophenetic correlation coefficient [44] and the clinical relevance of the final factorised matrices (which ensure model fit, stability and interpretability, respectively). The identification of trajectories using NMF requires person-time to be divided into discrete periods. In the present study, we selected a period of 1 year as offering an appropriate compromise between the sparsity of diagnosis data and an appropriate resolution at which the trajectories are updated.

This study was based on the WMC, a cohort derived from linked, routinely collected primary and secondary care data for the population of Wales. Although the WMC offers population-level coverage and enabled the ascertainment of a large number of acute and chronic conditions, there are a number of limitations associated with the use of the WMC that should be recognised. Firstly, despite the use of linked data, there still remains the possibility for disease misclassification (e.g. in the event of under- or misdiagnosis of a condition). However, our research produced a number of findings which were consistent with post-MI disease occurrences observed in other nationwide studies including in English and Danish populations [12, 27]. We expect disease misclassification was minimised by our use of an unrestricted look-back period (with at least 5 years of pre-MI data available for each individual) and the integration of data from both hospital- and community-based care providers [61]. This study was limited to individuals registered with a GP practice that contributed primary care data to the WMC. Including those individuals with secondary care data only would have led to systematic under-ascertainment of chronic conditions and biased trajectory allocation toward apparently ‘healthier’ profiles. We may therefore not capture some individuals in this study who are less likely to engage with healthcare services; however, given this was a small proportion of the overall population, we expect this to have had minimal impact on our findings. Data regarding behavioural risk factors, patient ethnicity and MI treatment was not available in the WMC, precluding evaluation of the association between these and post-MI disease trajectories. The definition of each condition studied was based on the presence or absence of CALIBER diagnosis codes and did not incorporate data regarding laboratory results or blood tests. The data available in the WMC does not differentiate between non-ST-elevation (NSTEMI) and ST-elevation myocardial infarction (STEMI), precluding analysis stratified by MI subtypes. Although the underlying ICD-10 codes for MI diagnosis were available within the SAIL databank, ICD-10 codes could not reliably differentiate MI subtype during the years included in the study period due to known differences in coding practices during that period [62]. Finally, although the National Health Service provides universal, publicly funded access to primary and secondary care in Wales, rural–urban variation in service utilisation and diagnostic opportunity may persist. Patients in remote areas may have reduced access to primary care and could therefore present with more advanced disease, leading to potential under-ascertainment of chronic conditions and reduced capture of multimorbidity, compared with urban patients. However, adjustment for rural–urban status, combined with linkage of both primary and secondary care records within SAIL, minimised the potential for bias.

### Future work

This study demonstrates the utility of non-negative matrix factorisation as a method for reducing the complexity of longitudinal clinical data into discrete time-varying trajectories. Alongside this work, we have released a package to implement NMF in Stata to further increase accessibility of the methods implemented here to epidemiologists and other applied clinical researchers [63]. Future work should focus on (i) establishing the reproducibility of the identified trajectories in similar populations of patients with MI, (ii) improving the a priori prediction of the specific trajectory at given patient will follow at a given time (e.g. at the time of MI, 2 years post-MI) to facilitate individualised risk prediction and (iii) identifying interventions or develop strategies that can modify the trajectory of an individual from a more adverse to a less adverse trajectory, in order to assess whether this may reduce the incidence of associated unfavourable outcomes. Given these data did not span the period of the COVID-19 pandemic, further work should also seek to ascertain the impact of infection with the COVID-19 virus on post-MI trajectories. An extension of this work would also be exploration of more sophisticated techniques of matrix factorisation, such as recently reported approaches to NMF that use deep artificial neural networks [64].

## Conclusions

In this nationwide study of patients diagnosed with acute myocardial infarction in Wales, we observed that patients with MI have a high pre-existing multimorbidity burden that increases further in the post-MI period. The application of NMF enabled the identification of ten latent multimorbidity trajectories that represent the complex clusters of acute and chronic conditions following MI over time. A number of these trajectories recapitulated patient groups that are observed clinically in the post-MI period—such as those that go on to develop gastrointestinal bleeding and anaemia (T_5_), those with diabetes-related issues (T_6_) and those with recurrent ischaemic-type presentations (T_2_ and T_10_). The most adverse trajectory, T_10_, was observed in 36.9% of the cohort (more commonly in older male patients) and characterised by recurrent MI, acute infections and renal disease. These trajectories remained associated with differential clinical outcomes following adjustment for age, sex and other confounders. The ability to objectively characterise such trajectories using routinely collected EHR data can enable the implementation of targeted strategies (such as enhanced surveillance or additional secondary preventative pharmacotherapy) for individuals that are at greatest risk of following these adverse courses in the post-MI period. Lastly, this work provides the necessary rigorous applied methodology for deepening our understanding of the complex trajectories of disease progression for other multimorbid populations.

## Supplementary Information

Additional file 1: Table S1. Summary of all acute and chronic conditions included in this study.

Additional file 2: Supplementary methods regarding the mathematical basis of NMF.

Additional file 3: Checklist S1. The RECORD checklist of items, extended from the STROBE statement, that should be reported in observational studies using routinely collected health data.

Additional file 4: Checklist S2.The CODE-EHR framework: checklist to report on the use of structured electronic healthcare records in clinical research.

Additional file 5: Fig. S1. Results of running NMF for values of k from 3 to 25. NMF was run a total of 10 times for each value of $k$. The 10-cluster solution was deemed to be optimal as it resulted in minima for model fit statistics (both Kullback–Leibler divergence and residual sum of squares) and resulted in optimal clinical interpretability of the resulting clusters. The cophenetic correlation coefficient had high inter-run variability and was deemed not useful in identifying k.

Additional file 6: Table S2. Validation of stability of identified trajectories. Cluster-wise Jaccard indices and corresponding 95% confidence intervals calculated using 1000 bootstrap replicates.

Additional file 7: Table S3. Multinomial regression analysis to identify sociodemographic characteristics associated with each single-year disease cluster.

## Data Availability

The data that support the findings of this study are available from the SAIL Databank, but restrictions apply to the availability of these data, which were used under license for the current study, and so are not publicly available. Data are however available with permission of the SAIL Databank. The code used to apply NMF is available at: https://github.com/jonathanbatty/nmf-trajectories-in-mi.

## Abbreviations

ICD-10: International Classification of Diseases, version 10
MI: Myocardial infarction
NMF: Non-negative matrix factorisation
NSTEMI: Non-ST-elevation myocardial infarction
PEDW: Patient Episode Database for Wales
SAIL: Secure Anonymised Information Linkage
STEMI: ST-elevation myocardial infarction
WLGP: Welsh Longitudinal General Practice dataset
WMC: Wales Multimorbidity e-Cohort
