# Supplementary material for: Long-term accrual of conditions following myocardial infarction: a study of disease trajectories in the Wales Multimorbidity e-Cohort
Source: BMC Med. 2025 Nov 26;23:710. doi: 10.1186/s12916-025-04520-1 (PMC12751190; doi:10.1186/s12916-025-04520-1)
Supplement: Supplementary file 1 — Additional file 1: Table S1. Summary of all acute and chronic conditions included in this study. [file 12916_2025_4520_MOESM1_ESM.docx]

**Table S1 |** Summary of all acute and chronic conditions included in this study, adapted from the CALIBER condition list.

| **#** | **Disease State** | **Inclusion / Chronicity** |
| --- | --- | --- |
| 1 | Abdominal aortic aneurysm | Chronic |
| 2 | Abdominal hernia | Chronic |
| 3 | Acne | Chronic |
| 4 | Actinic keratosis | Chronic |
| 5 | Acute kidney injury | Acute |
| 6 | Agranulocytosis | Acute |
| 7 | Alcohol problems | Chronic |
| 8 | Alcoholic liver disease | Chronic |
| 9 | Allergic and chronic rhinitis | Chronic |
| 10 | Alopecia areata | Chronic |
| 11 | Anal fissure | Acute |
| 12 | Angiodysplasia of colon | Chronic |
| 13 | Ankylosing spondylitis | Chronic |
| 14 | Anorectal fistula | Chronic |
| 15 | Anorectal prolapse | Chronic |
| 16 | Anorexia and bulimia nervosa | Chronic |
| 17 | Anterior and intermediate uveitis | Acute |
| 18 | Anxiety disorders | Chronic |
| 19 | Aplastic anaemias | Chronic |
| 20 | Appendicitis | Acute |
| 21 | Asbestosis | Chronic |
| 22 | Aspiration pneumonitis | Acute |
| 23 | Asthma | Chronic |
| 24 | Atrial fibrillation | Chronic |
| 25 | Atrioventricular block, complete | Chronic |
| 26 | Atrioventricular block, first degree | Chronic |
| 27 | Atrioventricular block, second degree | Chronic |
| 28 | Autism and Asperger's syndrome | Chronic |
| 29 | Autoimmune liver disease | Chronic |
| 30 | Bacterial diseases (excluding tuberculosis) | Acute |
| 31 | Bacterial sepsis of newborn | Excluded |
| 32 | Barrett's oesophagus | Chronic |
| 33 | Bell's palsy | Acute |
| 34 | Benign neoplasm and polyp of uterus | Chronic |
| 35 | Benign neoplasm of brain and other parts of central nervous system | Chronic |
| 36 | Benign neoplasm of colon, rectum, anus and anal canal | Chronic |
| 37 | Benign neoplasm of ovary | Chronic |
| 38 | Benign neoplasm of stomach and duodenum | Chronic |
| 39 | Bifascicular block | Chronic |
| 40 | Bipolar affective disorder and mania | Chronic |
| 41 | Bronchiectasis | Chronic |
| 42 | Carcinoma in situ, cervical | Chronic |
| 43 | Carpal tunnel syndrome | Chronic |
| 44 | Cataract | Chronic |
| 45 | Cerebral palsy | Chronic |
| 46 | Cholangitis | Acute |
| 47 | Cholecystitis | Acute |
| 48 | Cholelithiasis | Chronic |
| 49 | Chronic kidney disease | Chronic |
| 50 | Chronic sinusitis | Chronic |
| 51 | Chronic viral hepatitis | Chronic |
| 52 | Coeliac disease | Chronic |
| 53 | Collapsed vertebra | Chronic |
| 54 | Congenital malformations of cardiac septa | Chronic |
| 55 | Chronic obstructive pulmonary disease | Chronic |
| 56 | Coronary heart disease not otherwise specified | Excluded |
| 57 | Crohn's disease | Chronic |
| 58 | Cystic fibrosis | Chronic |
| 59 | Delirium, not induced by alcohol and other psychoactive substances | Acute |
| 60 | Dementia | Chronic |
| 61 | Depression | Chronic |
| 62 | Dermatitis (atopic/contact/other/unspecified) | Chronic |
| 63 | Diabetes | Chronic |
| 64 | Diabetic neurological complications | Chronic |
| 65 | Diabetic ophthalmic complications | Chronic |
| 66 | Diaphragmatic hernia | Chronic |
| 67 | Dilated cardiomyopathy | Chronic |
| 68 | Disorders of autonomic nervous system | Chronic |
| 69 | Diverticular disease of intestine (acute and chronic) | Chronic |
| 70 | Down's syndrome | Chronic |
| 71 | Dysmenorrhoea | Acute |
| 72 | Ear and upper respiratory tract infections | Acute |
| 73 | Encephalitis | Acute |
| 74 | End stage renal disease | Chronic |
| 75 | Endometrial hyperplasia and hypertrophy | Chronic |
| 76 | Endometriosis | Chronic |
| 77 | Enteropathic arthropathy | Chronic |
| 78 | Enthesopathies and synovial disorders | Chronic |
| 79 | Epilepsy | Chronic |
| 80 | Erectile dysfunction | Chronic |
| 81 | Essential tremor | Chronic |
| 82 | Eye infections | Acute |
| 83 | Fatty liver | Chronic |
| 84 | Female genital prolapse | Chronic |
| 85 | Female infertility | Excluded |
| 86 | Female pelvic inflammatory disease | Acute |
| 87 | Fibromatoses | Chronic |
| 88 | Folate deficiency anaemia | Chronic |
| 89 | Fracture of hip | Acute |
| 90 | Fracture of wrist | Acute |
| 91 | Gastritis and duodenitis | Acute |
| 92 | Gastro-oesophageal reflux disease | Chronic |
| 93 | Giant cell arteritis | Chronic |
| 94 | Glaucoma | Chronic |
| 95 | Glomerulonephritis | Chronic |
| 96 | Gout | Chronic |
| 97 | Haemangioma, any site | Chronic |
| 98 | Hearing loss | Chronic |
| 99 | Heart failure | Chronic |
| 100 | Hepatic failure | Chronic |
| 101 | Hidradenitis suppurativa | Chronic |
| 102 | High birth weight | Excluded |
| 103 | Human immunodeficiency virus | Excluded* |
| 104 | Hodgkin lymphoma | Chronic |
| 105 | Hydrocoele (including infected) | Chronic |
| 106 | Hyperkinetic disorders | Chronic |
| 107 | Hyperparathyroidism | Chronic |
| 108 | Hyperplasia of prostate | Chronic |
| 109 | Hypertension | Chronic |
| 110 | Hypertrophic cardiomyopathy | Chronic |
| 111 | Hypertrophy of nasal turbinates | Chronic |
| 112 | Hypo or hyperthyroidism | Chronic |
| 113 | Hyposplenism | Chronic |
| 114 | Immunodeficiencies | Chronic |
| 115 | Infection of anal and rectal regions | Acute |
| 116 | Infection of bones and joints | Acute |
| 117 | Infection of liver | Acute |
| 118 | Infection of male genital system | Acute |
| 119 | Infection of other or unspecified genitourinary system | Acute |
| 120 | Infection of skin and subcutaneous tissues | Acute |
| 121 | Infections of other or unspecified organs | Acute |
| 122 | Infections of the digestive system | Acute |
| 123 | Infections of the heart | Acute |
| 124 | Intellectual disability | Chronic |
| 125 | Intervertebral disc disorders | Chronic |
| 126 | Intracerebral haemorrhage | Acute |
| 127 | Intracranial hypertension | Chronic |
| 128 | Intrauterine hypoxia | Excluded |
| 129 | Iron deficiency anaemia | Chronic |
| 130 | Irritable bowel syndrome | Chronic |
| 131 | Ischaemic stroke | Chronic |
| 132 | Juvenile arthritis | Chronic |
| 133 | Keratitis | Acute |
| 134 | Left bundle branch block | Chronic |
| 135 | Leiomyoma of uterus | Chronic |
| 136 | Leukaemia | Chronic |
| 137 | Lichen planus | Chronic |
| 138 | Liver fibrosis, sclerosis and cirrhosis | Chronic |
| 139 | Low high density lipoprotein cholesterol | Excluded* |
| 140 | Lower respiratory tract infections | Acute |
| 141 | Lupus erythematosus (local and systemic) | Chronic |
| 142 | Macular degeneration | Chronic |
| 143 | Male infertility | Excluded |
| 144 | Meniere disease | Chronic |
| 145 | Meningitis | Acute |
| 146 | Menorrhagia and polymenorrhoea | Chronic |
| 147 | Migraine | Chronic |
| 148 | Monoclonal gammopathy of undetermined significance | Chronic |
| 149 | Motor neuron disease | Chronic |
| 150 | Multiple myeloma and malignant plasma cell neoplasms | Chronic |
| 151 | Multiple sclerosis | Chronic |
| 152 | Multiple valve disease | Chronic |
| 153 | Myasthenia gravis | Chronic |
| 154 | Mycoses | Acute |
| 155 | Myelodysplastic syndromes | Chronic |
| 156 | Myocardial infarction | Acute |
| 157 | Nasal polyp | Chronic |
| 158 | Neonatal jaundice (excluding haemolytic disease of the newborn) | Excluded |
| 159 | Neuromuscular dysfunction of bladder | Chronic |
| 160 | Non-acute cystitis | Chronic |
| 161 | Non-Hodgkin lymphoma | Chronic |
| 162 | Non-rheumatic aortic valve disorders | Chronic |
| 163 | Non-rheumatic mitral valve disorders | Chronic |
| 164 | Obesity | Chronic |
| 165 | Obsessive-compulsive disorder | Chronic |
| 166 | Obstructive and reflux uropathy | Chronic |
| 167 | Oesophageal varices | Chronic |
| 168 | Oesophagitis and oesophageal ulcer | Chronic |
| 169 | Osteoarthritis (excluding spine) | Chronic |
| 170 | Osteoporosis | Chronic |
| 171 | Other anaemias | Acute |
| 172 | Other cardiomyopathy | Chronic |
| 173 | Other haemolytic anaemias | Chronic |
| 174 | Other interstitial pulmonary diseases with fibrosis | Chronic |
| 175 | Other nervous system infections | Acute |
| 176 | Other or unspecified infectious organisms | Acute |
| 177 | Other psychoactive substance misuse | Chronic |
| 178 | Pancreatitis | Acute |
| 179 | Parasitic infections | Acute |
| 180 | Parkinson's disease | Chronic |
| 181 | Patent ductus arteriosus | Chronic |
| 182 | Peptic ulcer disease | Chronic |
| 183 | Pericardial effusion (non-inflammatory) | Chronic |
| 184 | Peripheral arterial disease | Chronic |
| 185 | Peripheral neuropathies (excluding cranial nerve and carpal tunnel syndromes) | Chronic |
| 186 | Peritonitis | Acute |
| 187 | Personality disorders | Chronic |
| 188 | Pilonidal cyst/sinus | Chronic |
| 189 | Pleural effusion | Acute |
| 190 | Pleural plaque | Chronic |
| 191 | Pneumothorax | Acute |
| 192 | Polycystic ovarian syndrome | Chronic |
| 193 | Polycythaemia vera | Chronic |
| 194 | Polymyalgia rheumatica | Chronic |
| 195 | Portal hypertension | Chronic |
| 196 | Post-coital and contact bleeding | Acute |
| 197 | Posterior Uveitis | Chronic |
| 198 | Post-infective and reactive arthropathies | Chronic |
| 199 | Postmenopausal bleeding | Chronic |
| 200 | Post-term infant | Excluded |
| 201 | Post-viral fatigue syndrome, neurasthenia and fibromyalgia | Chronic |
| 202 | Prematurity | Excluded |
| 203 | Primary malignancy, biliary tract | Chronic |
| 204 | Primary malignancy, bladder | Chronic |
| 205 | Primary malignancy, bone and articular cartilage | Chronic |
| 206 | Primary malignancy, brain, other central nervous system and intracranial | Chronic |
| 207 | Primary malignancy, breast | Chronic |
| 208 | Primary malignancy, cervical | Chronic |
| 209 | Primary malignancy, colorectal and anus | Chronic |
| 210 | Primary malignancy, kidney and ureter | Chronic |
| 211 | Primary malignancy, liver | Chronic |
| 212 | Primary malignancy, lung and trachea | Chronic |
| 213 | Primary malignancy, malignant melanoma | Chronic |
| 214 | Primary malignancy, mesothelioma | Chronic |
| 215 | Primary malignancy, multiple independent sites | Chronic |
| 216 | Primary malignancy, oesophageal | Chronic |
| 217 | Primary malignancy, oropharyngeal | Chronic |
| 218 | Primary malignancy, other organs | Chronic |
| 219 | Primary malignancy, other skin and subcutaneous tissue | Chronic |
| 220 | Primary malignancy, ovarian | Chronic |
| 221 | Primary malignancy, pancreatic | Chronic |
| 222 | Primary malignancy, prostate | Chronic |
| 223 | Primary malignancy, stomach | Chronic |
| 224 | Primary malignancy, testicular | Chronic |
| 225 | Primary malignancy, thyroid | Chronic |
| 226 | Primary malignancy, uterine | Chronic |
| 227 | Primary or idiopathic thrombocytopenia | Chronic |
| 228 | Primary pulmonary hypertension | Chronic |
| 229 | Psoriasis | Chronic |
| 230 | Psoriatic arthropathy | Chronic |
| 231 | Ptosis of eyelid | Chronic |
| 232 | Pulmonary collapse (excluding pneumothorax) | Acute |
| 233 | Pulmonary embolism | Chronic |
| 234 | Raised low density lipoprotein cholesterol | Excluded† |
| 235 | Raised total cholesterol | Excluded† |
| 236 | Raised triglycerides | Excluded† |
| 237 | Raynaud's syndrome | Chronic |
| 238 | Respiratory distress of newborn | Excluded |
| 239 | Respiratory failure | Acute |
| 240 | Retinal detachments and breaks | Acute |
| 241 | Retinal vascular occlusions | Acute |
| 242 | Rheumatic fever | Acute |
| 243 | Rheumatic valve disease | Chronic |
| 244 | Rheumatoid arthritis | Chronic |
| 245 | Right bundle branch block | Chronic |
| 246 | Rosacea | Chronic |
| 247 | Sarcoidosis | Chronic |
| 248 | Schizophrenia, schizotypal and delusional disorders | Chronic |
| 249 | Scleritis and episcleritis | Acute |
| 250 | Scoliosis | Chronic |
| 251 | Seborrheic dermatitis | Chronic |
| 252 | Secondary malignancy, adrenal gland | Chronic |
| 253 | Secondary malignancy, bone | Chronic |
| 254 | Secondary malignancy, bowel | Chronic |
| 255 | Secondary malignancy, brain, other central nervous system and intracranial | Chronic |
| 256 | Secondary malignancy, liver and intrahepatic bile duct | Chronic |
| 257 | Secondary malignancy, lung | Chronic |
| 258 | Secondary malignancy, lymph nodes | Chronic |
| 259 | Secondary malignancy, other organs | Chronic |
| 260 | Secondary malignancy, pleura | Chronic |
| 261 | Secondary malignancy, retroperitoneum and peritoneum | Chronic |
| 262 | Secondary or other thrombocytopenia | Chronic |
| 263 | Secondary polycythaemia | Chronic |
| 264 | Secondary pulmonary hypertension | Chronic |
| 265 | Septicaemia | Acute |
| 266 | Sick sinus syndrome | Chronic |
| 267 | Sickle-cell anaemia | Chronic |
| 268 | Sickle-cell trait | Chronic |
| 269 | Sjogren's disease | Chronic |
| 270 | Sleep apnoea | Chronic |
| 271 | Slow foetal growth or low birth weight | Excluded |
| 272 | Spina bifida | Chronic |
| 273 | Spinal stenosis | Chronic |
| 274 | Splenomegaly | Chronic |
| 275 | Spondylolisthesis | Chronic |
| 276 | Spondylosis | Chronic |
| 277 | Stable angina | Chronic |
| 278 | Stroke, not otherwise specified | Chronic |
| 279 | Subarachnoid haemorrhage | Acute |
| 280 | Subdural haematoma, non-traumatic | Chronic |
| 281 | Supraventricular tachycardia | Acute |
| 282 | Syndrome of inappropriate secretion of antidiuretic hormone | Chronic |
| 283 | Systemic sclerosis | Chronic |
| 284 | Thalassaemia | Chronic |
| 285 | Thalassaemia trait | Chronic |
| 286 | Thrombophilia | Chronic |
| 287 | Tinnitus | Chronic |
| 288 | Transient ischaemic attack | Acute |
| 289 | Trifascicular block | Chronic |
| 290 | Trigeminal neuralgia | Acute |
| 291 | Tuberculosis | Acute |
| 292 | Tubulo-interstitial nephritis | Acute |
| 293 | Ulcerative colitis | Chronic |
| 294 | Undescended testicle | Excluded |
| 295 | Unstable angina | Acute |
| 296 | Urinary incontinence | Chronic |
| 297 | Urinary tract infection | Acute |
| 298 | Urolithiasis | Chronic |
| 299 | Urticaria | Acute |
| 300 | Venous thromboembolic disease (excluding pulmonary embolism) | Chronic |
| 301 | Ventricular tachycardia | Acute |
| 302 | Viral diseases (excluding chronic hepatitis or human immunodeficiency virus) | Acute |
| 303 | Visual impairment and blindness | Chronic |
| 304 | Vitamin B12 deficiency anaemia | Chronic |
| 305 | Vitiligo | Chronic |
| 306 | Volvulus | Acute |

Notes: *Diagnostic codes were not used to derive an HIV phenotype in the eWMC, in line with SAIL policies. †These CALIBER disease states were not ascertained in the eWMC.
