# Supplementary material for: Long-term accrual of conditions following myocardial infarction: a study of disease trajectories in the Wales Multimorbidity e-Cohort
Source: BMC Med. 2025 Nov 26;23:710. doi: 10.1186/s12916-025-04520-1 (PMC12751190; doi:10.1186/s12916-025-04520-1)
Supplement: Supplementary file 2 — Additional file 2: Supplementary methods regarding the mathematical basis of NMF. [file 12916_2025_4520_MOESM2_ESM.docx]

Supplementary Methods

*The mathematics of non-negative matrix factorisation (NMF)*

Briefly, NMF refers to a group of algorithms that decompose a matrix, $A$ (of dimensions $u$row $\times v$ columns) into two lower-rank, non-negative matrices $W$ ($u\times k$) and $H$ ($k\times v$), whereby $A\approx WH$, $W\geq0$ and $H\geq0$ and the rank of the decomposition, $k\ll A$[33]. In reducing the dimensionality of a matrix, NMF has an inherent clustering property. The non-negativity of the resulting matrices makes these directly interpretable and amenable to subsequent statistical analysis. ss

A data matrix, $A\in\mathbb{R}^{m\times n}$, representing patient-time ($m$ rows) × diagnoses ($n$ columns) can be approximated using the matrix factorisation:

$$\begin{aligned} A\approx WH^{\top}\#\left( 1 \right) \end{aligned}$$

Where $W=\left[ w_{1},\ldots, w_{k} \right]\in\mathbb{R}^{m\times k}$ and $H^{\top}=\left[ h_{1},\ldots, h_{k} \right]\in\mathbb{R}^{n\times k}$ are the factor matrices. These summarise the original patient-time × diagnosis matrix using $k$ components, with $k\ll min\left\{ m,n \right\}$.

Once the patient-time × diagnosis matrix ($A$) is factorised, the factor matrix $W$ represents the degree of expression of each of $k$ disease clusters at a given time point. The factor matrix $H$ represents the degree of membership of each disease in each of $k$ disease clusters. Therefore, the dimensionality of disease states has been reduced to $k$; disease states have effectively been ‘clustered’ into $k$ phenotypes.

The factor matrices $W$ and $H$ may be computed from the patient-time × diagnosis matrix ($A$) by solving the following optimisation problem:

$$\begin{aligned} {\min_{W,H\geq0} \left\| A-WH \right\|}_{KL}\#\left( 2 \right) \end{aligned}$$

Where $\left\| \cdot\right\|_{KL}$ denotes the generalised Kullback-Leibler Divergence (also referred to as the I-divergence), $D_{KL}\left( A||\hat{A} \right)=\sum_{i,j} \left( A_{i,j}\log\frac{A_{i,j}}{\hat{A}_{i,j}}-A_{ij}+\hat{A}_{ij} \right)$, for $i,j>0$. The Kullback-Leibler divergence has a Poisson distribution and is preferred over a least-squares-based loss function when $A$ is composed of sparse indicator or count data, such as the case with EHR-derived diagnosis data.

NMF based on the Kullback-Leibler divergence can be computed using component-wise multiplicative update rules. Values of one of the factor matrices, $W$ or $H$, are updated while keeping the other fixed, which leads to a decrease in the objective function, as follows[41]:

$$\begin{aligned} H\leftarrow H\circ\frac{W^{T}\frac{X}{WH}}{W^{T}}\#\left( 3 \right) \end{aligned}$$

$$\begin{aligned} W\leftarrow W\circ\frac{\frac{X}{WH}H^{T}}{H^{T}}\#\left( 4 \right) \end{aligned}$$

Note that the NMF process is nondeterministic: the factor matrices $W$ and $H$ are initialised with random starting values. During the multiplicative updating process, local minima can be encountered. As such, it is necessary to run the NMF process multiple times in order to improve the likelihood that a true global minimum is determined. NMF is a nondeterministic polynomial-time hard (NP-hard) computational problem, with a computational complexity of $O(mnk)$ per iteration.

*Interpretation of decomposed matrices (W and H)*

Matrix $W$ represents the ‘expression’ of each of $k$ *disease clusters* for an individual in each discrete time period, and matrix $H$ represents the membership of individual conditions in each cluster. The numbers included in each matrix are non-missing and non-negative, but do not have a direct, unit-based interpretation. The values in matrix $W$ represent the extent to which an person-year of time belongs to a given disease cluster, *relative to* the degree of membership to the other disease clusters included in that row. Similarly, the values in matrix $H$ represent the extent to which each disease features in a given disease cluster, *relative to* all other diseases. The actual numbers in the matrices are unitless and do not have a direct interpretation in isolation.

*Enumeration of optimal matrix factorisation* ($k$)

In order to select the value of $k$ (and therefore the number of disease clusters) that best model the person-time diagnosis data, the residual Kullback-Leibler Divergence and sum of squares (RSS) error between the original matrix, $A$ and the product of $WH$ was plotted. The minimum error obtained from performing 10 replicates of NMF for each value of $k$ between 3 and 25 was plotted (Supplementary Figure 2). The inflection point (or “knee”) was identified as the point of maximal curvature of the resulting curve, as identified using the *Kneedle* package in Python[43]. Briefly, the knee-finding algorithm: (i) normalises the curve data and fits a smoothing spline function, (ii) calculates the distance between each point of the curve and a straight line connecting the first and last points of the curve, and (iii) identifies the point of the curve at the greatest distance from the line. This was supplemented by evaluating the cophenetic correlation coefficient (a measuring of the stability of matrix decomposition across multiple runs) and examining the clinical interpretability of the factorisation solution[44].
