# Supplementary figures and images for: Long-term accrual of conditions following myocardial infarction: a study of disease trajectories in the Wales Multimorbidity e-Cohort
Source: BMC Med. 2025 Nov 26;23:710. doi: 10.1186/s12916-025-04520-1 (PMC12751190; doi:10.1186/s12916-025-04520-1)

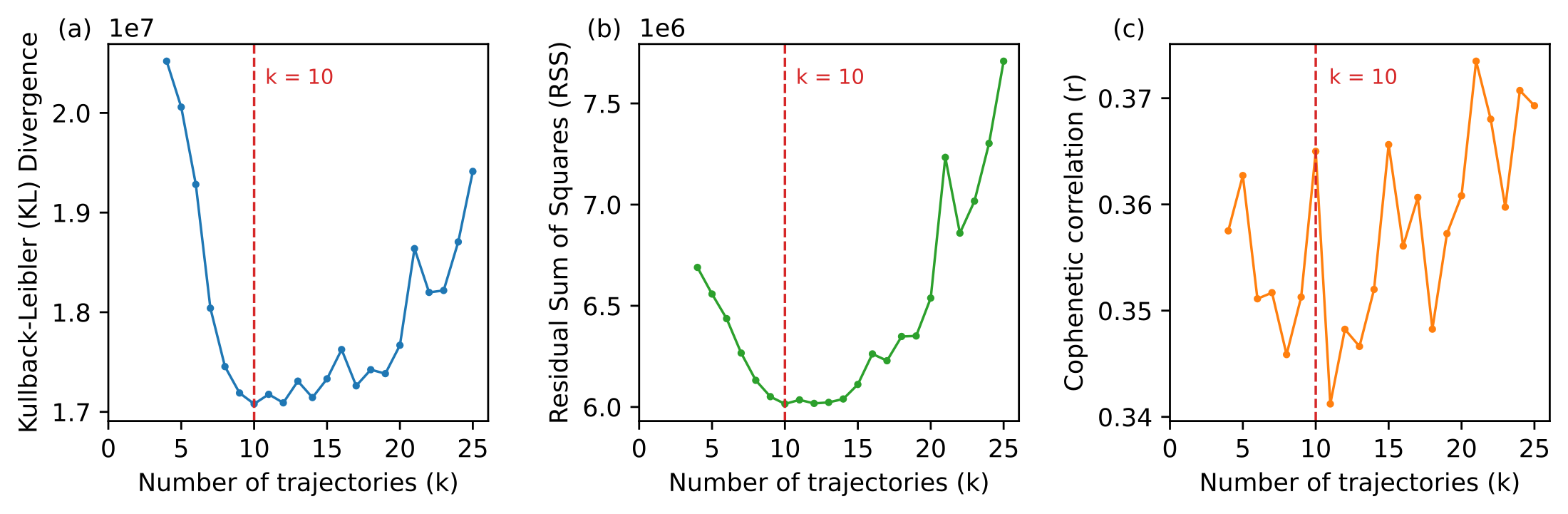

Supplement: Supplementary file 5 — Additional file 5: Fig. S1. Results of running NMF for values of k from 3 to 25. NMF was run a total of 10 times for each value of $k$. The 10-cluster solution was deemed to be optimal as it resulted in minima for model fit statistics (both Kullback–Leibler divergence and residual sum of squares) and resulted in optimal clinical interpretability of the resulting clusters. The cophenetic correlation coefficient had high inter-run variability and was deemed not useful in identifying k. [file 12916_2025_4520_MOESM5_ESM.tif]
