# Supplementary material for: Long-term accrual of conditions following myocardial infarction: a study of disease trajectories in the Wales Multimorbidity e-Cohort
Source: BMC Med. 2025 Nov 26;23:710. doi: 10.1186/s12916-025-04520-1 (PMC12751190; doi:10.1186/s12916-025-04520-1)
Supplement: Supplementary file 6 — Additional file 6: Table S2. Validation of stability of identified trajectories. Cluster-wise Jaccard indices and corresponding 95% confidence intervals calculated using 1000 bootstrap replicates. [file 12916_2025_4520_MOESM6_ESM.docx]

**Table S2 |** Validation of stability of identified trajectories

| **Trajectory** | **Jaccard Index (95% confidence interval)** |
| --- | --- |
| 1 | 0.91 (0.81 – 1.00) |
| 2 | 0.79 (0.69 – 0.90) |
| 3 | 0.77 (0.68 – 0.86) |
| 4 | 0.70 (0.61 – 0.79) |
| 5 | 0.72 (0.62 – 0.81) |
| 6 | 0.80 (0.70 – 0.90) |
| 7 | 0.75 (0.65 – 0.84) |
| 8 | 0.83 (0.74 – 0.93) |
| 9 | 0.87 (0.77 – 0.98) |
| 10 | 0.95 (0.86 – 1.00) |

Cluster-wise Jaccard Indices and corresponding 95% confidence intervals were calculated using 1,000 bootstrap replicates
