# Supplementary material for: Long-term accrual of conditions following myocardial infarction: a study of disease trajectories in the Wales Multimorbidity e-Cohort
Source: BMC Med. 2025 Nov 26;23:710. doi: 10.1186/s12916-025-04520-1 (PMC12751190; doi:10.1186/s12916-025-04520-1)
Supplement: Supplementary file 7 — Additional file 7: Table S3. Multinomial regression analysis to identify sociodemographic characteristics associated with each single-year disease cluster. [file 12916_2025_4520_MOESM7_ESM.docx]

**Table S3 |** Multinomial regression analysis to identify sociodemographic characteristics associated with each single-year disease cluster.

| **Predictor** | **Relative risk ratio (RRR) and corresponding 95% CI for association between each socio-demographic factor and post-MI disease cluster** | | | | | | | | | |
| --- | --- | --- | --- | --- | --- | --- | --- | --- | --- | --- |
|  | **C_1_** | **C_2_** | **C_3_** | **C_4_** | **C_5_** | **C_6_** | **C_7_** | **C_8_** | **C_9_** | **C_10_** |
| Sex of patient | Base outcome | - - - | - - - | - - - | - - - | - - - | - - - | - - - | - - - | - - - |
| Male | Base outcome | Ref | Ref | Ref | Ref | Ref | Ref | Ref | Ref | Ref |
| Female | Base outcome | 1.23 (1.19 – 1.27) | 1.21 (1.16 – 1.26) | 0.92 (0.88 – 0.95) | 0.95 (0.92 – 0.99) | 0.74 (0.71 – 0.76) | 0.65 (0.63 – 0.67) | 0.66 (0.64 – 0.69) | 0.88 (0.85 – 0.90) | 0.54 (0.52 – 0.55) |
| WIMD | Base outcome | - - - | - - - | - - - | - - - | - - - | - - - | - - - | - - - | - - - |
| 1 – most deprived | Base outcome | Ref | Ref | Ref | Ref | Ref | Ref | Ref | Ref | Ref |
| 2 | Base outcome | 1.16 (1.11 – 1.21) | 1.04 (0.98 – 1.09) | 1.05 (1.00 – 1.11) | 1.10 (1.05 – 1.16) | 1.16 (1.10 – 1.21) | 1.19 (1.15 – 1.24) | 1.07 (1.02 – 1.12) | 1.02 (0.98 – 1.06) | 1.10 (1.05 – 1.15) |
| 3 | Base outcome | 1.24 (1.19 – 1.30) | 0.95 (0.90 – 1.00) | 1.11 (1.05 – 1.17) | 1.12 (1.06 – 1.17) | 1.28 (1.22 – 1.35) | 1.32 (1.27 – 1.37) | 1.04 (0.99 – 1.09) | 1.03 (0.99 – 1.07) | 1.15 (1.10 – 1.21) |
| 4 | Base outcome | 1.36 (1.30 – 1.42) | 0.95 (0.89 – 1.00) | 1.16 (1.10 – 1.23) | 1.11 (1.05 – 1.17) | 1.35 (1.28 – 1.41) | 1.42 (1.36 – 1.48) | 1.05 (1.00 – 1.11) | 1.00 (0.96 – 1.05) | 1.18 (1.12 – 1.24) |
| 5 – least deprived | Base outcome | 1.31 (1.25 – 1.37) | 0.78 (0.73 – 0.83) | 1.07 (1.01 – 1.13) | 1.31 (1.24 – 1.38) | 1.34 (1.28 – 1.41) | 1.41 (1.35 – 1.47) | 0.94 (0.90 – 0.99) | 0.97 (0.69 – 1.05) | 1.24 (1.19 – 1.31) |
| Age at MI (per year) | Base outcome | 1.03 (1.02 – 1.03) | 1.07 (1.06 – 1.07) | 1.07 (1.06 – 1.07) | 1.12 (1.12 – 1.13) | 1.20 (1.20 – 1.21) | 1.16 (1.16 – 1.17) | 1.10 (1.10 – 1.11) | 1.50 (1.49 – 1.51) | 1.10 (1.10 – 1.11) |
| Age in period (per year) | Base outcome | 1.02 (1.02 – 1.02) | 1.01 (1.01 – 1.02) | 1.01 (1.01 – 1.02) | 1.00 (0.99 – 1.00) | 0.90 (0.90 – 0.91) | 0.89 (0.89 – 0.90) | 0.96 (0.95 – 0.96) | 0.72 (0.72 – 0.73) | 0.98 (0.97 – 0.98) |

Abbreviations: CI – confidence interval, Ref – reference category, RRR – relative risk ratio.
